# Frequency and Risk Factors for Acute Kidney Injury in patients with COVID-19

**DOI:** 10.12669/pjms.38.4.4980

**Published:** 2022

**Authors:** Muhammad Anees, Omair Farooq, Muhammad Raza, Asim Mumtaz

**Affiliations:** 1 Prof. Muhammad Anees, MBBS, FCPS (Nephrology). Consultant Nephrologist, Farooq Hospital, West Wood Branch, Lahore, Pakistan; 2 Dr. Omair Farooq, MBBS, MRCP. Consultant Physician, Farooq Hospital, West Wood Branch, Lahore, Pakistan; 3 Dr. Muhammad Raza, MBBS. Medical Officer, Farooq Hospital, West Wood Branch, Lahore, Pakistan; 4 Prof. Asim Mumtaz, MBBS, MPhil (Chemical Pathology). Consultant Chemical Pathologist, Farooq Hospital, West Wood Branch, Lahore, Pakistan

**Keywords:** AKI, COVID-19, Hypoxemia, Increasing age, Inflammatory markers, Prolonged hospital stay

## Abstract

**Objective:**

To determine the frequency of Acute Kidney Injury (AKI) and its underlying risk factors in patients with Coronavirus Disease (COVID-19).

**Methods:**

This retrospective study was conducted by reviewing the medical records of patients admitted in Covid-19 Intensive Care Unit (ICU) of Farooq Hospital, West Wood Branch, Lahore during the period from 1^st^ April, 2020 to 30^th^ June, 2020. COVID-19 was diagnosed on basis of Real Time Polymerase Chain Reaction (RT-PCR) through nasal swab. Demographic, clinical and laboratory data were collected at the time of admission in the hospital. AKI was diagnosed on basis of ≥ 0.3 mg/dl increase in serum Creatinine (sCr) from baseline during the hospital stay. The outcome of study was AKI.

**Results:**

One hundred and seventy-six patients who fulfilled the inclusion criteria were recruited of which most were males (78.4%). The mean age was 51.26 ± 15.20 years and the frequency of AKI was 51.1%. The risk factors for AKI were increasing age (OR=2.10, *p*=0.017); presence of COVID-19 symptoms (OR=6.62, *p*=0.004); prolonged hospital stay (OR=2.26, *p*=0.011); Diabetes Mellitus (OR=1.81, *p*=0.057); hypoxemia (OR=5.98, *p*=0.000); leukocytosis (OR=2.91, *p*=0.002); lymphopenia (OR=5.77, *p*=0.000); hypoalbuminemia (OR=4.94, *p*=0.000); elevated C-reactive protein (CRP) (OR=6.20, *p*=0.000) and raised D-diamers (OR=3.16, *p*=0.000).

**Conclusions:**

AKI was present in half of the COVID-19 patients. The most significant risk factors for AKI were increasing age, prolonged hospital stay, hypoxemia, hypoalbuminemia, DM and raised inflammatory markers.

## INTRODUCTION

COVID-19 is a medical condition caused by a novel Coronavirus now called Severe Acute Respiratory Syndrome Coronavirus 2 (SARS-CoV-2) that was first identified after an outbreak of pneumonia in Wuhan, December, 2019.^1^ Later on, COVID-19 was declared as pandemic by the World Health Organization (WHO) on March 11, 2020.^2^ Over past one and half year, COVID-19 has spread to almost all over the world. In Pakistan, the first case of COVID-19 was reported in Karachi on February 26, 2020 and since then it has grown so exponentially that the total number of confirmed cases has already crossed over 398,024 in March, 2021.^3^

COVID-19 primarily involves respiratory system but can also encompass cardiovascular, gastrointestinal, renal and other systems.^4^ AKI is now common in hospitalized patients with COVID-19 but its incidence still varies greatly from 0.5% to 80.3%.^5^ Renal involvement in COVID-19 can manifest as Proteinuria, Hematuria, Glomerulonephritis, electrolyte disturbances and AKI. AKI has been shown to be multifactorial involving direct viral insult, indirect injury by sepsis, hemodynamic disturbances, cytokine storm, disseminated intravascular coagulation and some other unknown mechanisms.^6^ Underlying co-morbid conditions like increasing age, Diabetes Mellitus (DM), Hypertension (HTN), Chronic Kidney Disease (CKD) and Anaemia can predispose to AKI in COVID-19 patients.^7^

A study by Chan et al reported that 65% of patients who developed AKI underwent complete resolution, 35% progressed to Acute Kidney Disease (AKD) requiring temporary dialysis in 19% and rest of them ended up in End Stage Renal Disease (ESRD) requiring permanent renal replacement therapy (RRT).^8^ In Pakistan, according to author’s view, there was no data available regarding the incidence of AKI and risk factors associated with its development in patients with COVID 19, so this study was conducted to explore these neglected segments.

## METHODS

After approval from the Institutional Review Board (IRB) dated 20/03/21, IRB No. 0134, the study was carried out at Isolation, Intensive Care Unit (ICU) of Farooq Hospital, Westwood Branch, Lahore, which is 220 bedded modern healthcare center. Available medical records of all the patients hospitalized with COVID-19 from 1^st^ April 2020 to 30^th^ June, 2020 were retrospectively reviewed. The patients with confirmed diagnosis of COVID-19 by RT-PCR were included while the patients with history of CKD, pregnant females and patients with incomplete medical records were excluded from the study. Demographic and clinical data including age, gender, co-morbidities, presenting symptoms of COVID-19, vital signs at admission, duration of stay in the hospital, travel and family contact history for COVID-19 were recorded. Laboratory data including haematological parameters such as Haemoglobin level, Total Leukocyte, Neutrophils and Lymphocytes counts along with biochemical parameters like serum Urea and Creatinine, albumin and inflammatory markers (CRP and D dimers) were also taken to establish the severity of COVID-19. Based on sCr level, the diagnosis of AKI was made according to the Kidney Diseases Initiative Global Outcome (KDIGO) AKI guidelines.^9^ AKI was defined as an increase of ≥0.3 mg/dL in sCr from baseline during whole period of stay in the hospital. Stage 1, stage 2 and stage 3 AKI were defined respectively as an increase of 1.5 to 1.9, 2.0 to 2.9 and 3 or more folds in sCr level from baseline during whole period of stay in the hospital. Baseline sCr was defined as the minimum value of sCr during whole period of stay in the hospital. The outcome of the study was AKI.

The data was entered and analyzed using IBM-SPSS V-23. The continuous variables were described as Mean ± SD and categorical variables as frequency and percentages. AKI and Non-AKI subjects were compared using Student’s t-test. Odds ratios were also calculated. Multivariate analysis was done using Logistic Regression (backward elimination method) to determine significant predictors of AKI. A *p-*value <5% was taken for statistical significance.

## RESULTS

Medical records of 270 patients admitted during the study period were reviewed. One hundred and seventy-six patients who fulfilled the inclusion criteria were recruited in the study of which 138 (78.4%) were males. The mean age of the patients was 51.26±15.20 years and 94 (53.4%) patients had an age of 50 years or above. Only 5 (2.8%) patients had a positive history of travel and 19 (10.8%) had positive family contact history for COVID-19. Respiratory symptoms were present in 157 (89.2%) patients among which, 95 (54%) patients had serious, 44 (25%) had most common and 18 (10.2%) had fewer common symptoms of COVID-19. The average duration of hospital stay was eight days ranging from one to twenty days. DM was the most common co-morbidity seen in 70 (39.8%) patients. AKI was found in 90 (51.1%) hospitalized patients. We found 77/176(43.8%) patients had stage 1, 11/176 (6.3%) had stage 2 and 2/176 (1.1%) patients had stage 3 AKI respectively. The demographic, clinical and laboratory parameters affecting AKI in COVID-19 patients are shown in Table-I. Multivariate analysis using Logistic Regression showed statistically significant factors affecting AKI were hypoxemia (*p*=0.000) and raised inflammatory markers at the time of admission (D dimers (*p*=0.007)) along with prolonged hospital stay (*p*=0.026). Using the univariate analysis statistically significant risk factors for developing AKI were defined in Table II. The overall mortality was 5.1% and patients having AKI were having high mortality than non AKI patients (3.4% Vs 1.7%) as shown in Table-III.

**Table-I:** Demographic, Clinical and Laboratory parameters as Risk Factors for developing AKI.

S. No.	Variables	Overall (N=176)	AKI (N=90)	Non-AKI (N=86)	p value AKI vs. Non-AKI
1	Age (years)	51.26 ± 15.20	55.49 ± 12.02	46.83 ± 16.90	0.000*
2	Duration of hospital stay (days)	8.46 ± 4.12	9.34 ± 3.60	7.53 ± 4.44	0.003*
3	Respiratory Rate (breaths/min)	19.77 ± 4.27	20.84 ± 3.89	18.64 ± 4.38	0.001*
4	Oxygen Saturation (%)	93.08 ± 5.03	90.82 ± 5.11	95.44 ± 3.70	0.000*
5	Haemoglobin (g/dL)	13.43 ± 1.66	13.41 ± 1.78	13.46 ± 1.54	0.869
6	Total Leukocyte Count (X 10^3^/µL)	9.16 ± 3.75	10.15 ± 4.11	8.13 ± 3.03	0.000*
7	Neutrophils (%)	71.43 ± 12.04	76.66 ± 9.95	66.95 ± 11.63	0.000*
8	Lymphocytes (%)	22.33 ± 11.00	17.72 ± 8.57	27.16 ± 11.24	0.000*
9	Serum Urea at admission (mg/dL)	42.94 ± 35.90	53.50 ± 43.26	31.90 ± 21.28	0.000*
10	Serum Creatinine at admission (mg/dL)	1.18 ± 0.64	1.34 ± 0.85	1.01 ± 0.20	0.001*
11	Serum Creatinine maximum (mg/dL)	1.40 ± 0.93	1.73 ± 1.19	1.05 ± 0.20	0.000*
12	Serum Albumin (g/dL)	3.82 ± 0.58	3.58 ± 0.45	4.08 ± 0.58	0.000*
13	CRP (mg/L)	65.62 ± 61.75	84.14 ± 59.70	46.23 ± 58.10	0.000*
14	D-diamers (µg/ml)	1.17 ± 1.62	1.53 ± 1.91	0.78 ± 1.14	0.002*

* p value is of statistical significance.

**Table-II:** Unadjusted Odds Ratios for developing AKI among COVID-19 patients.

S. No.	Variables	Unadjusted OR^1^	95% CI^2^	p value
1	Increasing age (>50 years)	2.10	1.14 – 3.87	0.017*
2	Gender (Male)	1.21	0.59 – 2.48	0.600
3	Prolong hospital Stay (>7 Days)	2.26	1.20 – 4.22	0.011*
4	Symptoms of COVID-19	6.62	1.85 – 23.66	0.004*
5	Diabetes Mellitus	1.81	0.98 – 3.34	0.057
6	Tachypnoea (>20 breaths/minute)	5.47	2.86 – 10.47	0.000*
7	Hypoxemia (<93%)	5.98	3.08 – 11.58	0.000*
8	Anaemia (<13 g/dL)	0.76	0.41 – 1.40	0.388
9	Leukocytosis (>11,000/µL)	2.91	1.46 – 5.80	0.002*
10	Neutrophilia (>70%)	6.56	0.31 – 12.99	0.000*
11	Lymphopenia (<30%)	5.77	2.47 – 13.50	0.000*
12	Hypoalbuminemia (< 3.5 g/dL)	4.94	2.53 – 9.61	0.000*
13	High Serum Creatinine (>1.1 mg/dL)	2.91	1.58 – 5.37	0.001*
14	Elevated C-reactive protein (>5mg/L)	6.20	2.23 – 17.22	0.000*
15	Raised D dimers (>0.5 µg/ml)	3.16	1.68 – 5.94	0.000*

OR: Odds Ratio, CI: confidence interval,

* p value of statistical significance

**Table-III:** A comparison of incidence of AKI and its Risk Factors and associated mortality in COVID-19 among different studies.

S. No.	Factors	Present Study (Pakistan) N = 176	Aparicio et al (Mexico)^13^ N = 99	Fisher et al (USA) 10 N = 4610	Chan et al (USA)^8^ N = 3993	Philips et al (UK)^15^ N = 632	See et al (Singapore)^7^ N = 707
1	Incidence of AKI (%)	51.1	58.6	56.9	46	34.2	8.1
2	Increasing age (years)	0.000*	0.010*	0.001*	0.001*	0.005*	0.005*
3	DM	0.057*	0.110	NA	0.000*	0.001*	0.005*
4	Male (%)	0.600 (78.4)	0.810 (74.7)	0.001* (57.3)	NA (57.3)	0.218 (57.4)	NA (57)
5	Prolonged Hospital Stay (days)	0.003*	0.02*	0.001*	NA	0.001*	0.005*
6	Hypoxemia (%)	0.000*	NA	0.001*	0.001*	NA	0.000*
7	High Creatinine (mg/dL)	0.001*	0.01*	NA	0.001*	NA	0.005*
8	Leukocytosis (X10^3^/µL)	0.000*	0.34	0.001*	0.001*	0.001	0.000*
9	Lymphopenia(%)	0.000*	0.010*	0.400	0.001*	0.065	0.005*
10	Hypoalbuminemia (g/dL)	0.000*	0.010*	NA	0.001*	0.001*	NA
11	High FDPS (µg/ml)	0.002*	0.010*	0.001*	NA	0.001*	NA
12	High CRP (mg/L)	0.000*	0.030*	0.001*	NA	0.001*	0.005*
13	Mortality % (AKI Vs Non-AKI)	3.4 Vs 1.7	65.5 Vs 14.6	33.7 Vs 9.3	50 Vs 8	50 Vs 21.1	12 Vs 1

* p value of statistical significance

## DISCUSSION

COVID-19 is primarily a respiratory illness affecting lungs but in severe disease, it can also involve other systems like kidneys, heart and liver thus leading to their impairment. In this study, almost half of the patients were found to have AKI. The results were consistent with the findings from other studies which reported a similar incidence ranging from 46% to 56.9%.^8^,^10^ In contrast, a much lower incidence ranging from 5% to 15%^11^ along with a pooled incidence of 10%^12^ previously had also been reported supporting that the exact incidence of AKI in COVID-19 is still to be determined. A comparatively higher incidence of AKI in local setting was due to: non serious attitude of public towards COVID19 during initial days of pandemic because of lack of awareness; reluctance from reporting to hospitals; reliance on home based treatment thus leading to very late presentation of patients in critical state at Accident and Emergency departments of hospitals after failed home management; availability of limited number of hospitals in Government sector catering COVID-19 providing poor health care services and lack of universal access to private sector health care facilities because of financial limitations. As the consequence, most of the patients in this study had a very late presentation in Accident and Emergency Department of the hospital, with serious symptoms, hypoxemia and tachypnoea. Later on, most of these patients developed AKI during hospital stay.

AKI in COVID-19 is multifactorial involving direct viral invasion or indirect ischemic or septic insult to renal tubular epithelium causing ATN. In this study, COVID-19 patients with AKI were found to have statistically significant higher values of all inflammatory markers as supported by other studies.^13^,^14^ Pathogenesis may include viral induced recruitment of neutrophils leading to Neutrophilia and Leukocytosis along with viral mediated damage followed by apoptosis of lymphocytes causing Lymphopenia.^14^ In severe SARS-CoV-2 infection with sepsis, Cytokine Release Syndrome (CRS) characterized by sudden massive release of pro-inflammatory cytokines and Hyper-inflammatory Syndrome characterized by elevated serum Levels of critical inflammatory mediators like CRP and D-dimers may result in Acute Respiratory Distress Syndrome (ARDS), respiratory, hepatic, renal failure and even death.

COVID-19 patients who are older, males, malnourished or diabetic are more prone to develop AKI.^15^ In current study, patients with higher age had AKI more than patients with relatively lower age as also reported by other studies.^10^,^13^,^15^ This may be due to immunocompromised elderly people are more likely to develop COVID-19 often along with an atypical presentation and complications making diagnosis and management difficult.^16^ Apart from COVID-19, age related structural and functional changes in kidneys leading to reduced renal mass, functioning nephron number and baseline kidney function along with increased likelihood of dehydration, reno-vascular occlusion and impairment of renal auto-regulation also make elderly people highly vulnerable to AKI.^17^

COVID-19 patients having DM are at increased risk of developing AKI as compared to non-diabetics. In present study, DM came out to be a significant risk factor for developing AKI as reported by other studies.^10^,^13^,^15^ In a study by Khalili et al.^18^, amongst hospitalized COVID-19 patients, AKI developed more in diabetics as compared to non-diabetics (62% vs. 38%). Hyperglycemia, suppressed immunity, thromboembolic events and sepsis/septic shock are important pathogenic mechanisms involved in causing AKI in DM.^19^-^21^ DM itself is an independent risk factor for AKI in general population.

According to medical literature,^8^,^13^,^15^ COVID-19 patients with hypoalbuminemia are more likely to develop AKI than patients with normal serum albumin level as observed in this study as well. An increased susceptibility to COVID-19 because of decreased immunity^22^ along with intravascular volume depletion resulting from extravasation of fluid are responsible for causing AKI in COVID-19 patients with hypoalbuminemia. A meta-analysis of 168,740 subjects concluded that each 1.0 g/dL decrement of serum albumin levels was associated with a 1.7-fold increased risk of AKI.^23^

COVID-19 patients with AKI have significantly prolonged hospital stay than patients without AKI as in current study and also supported by previous studies.^10^,^13^,^15^ This is probably because of patients presenting late in a critical state with AKI require prolonged hospital admission. COVID-19 patients have a high (39%) all-cause mortality.^24^ Saeed et al. reported a mortality rate of 70.7% amongst COVID-19 patients who developed AKI.^25^ In current study, overall mortality was low (5.1%) but three fourth of the patients died had AKI. Results were consistent with previous studies.^8^,^10^,^13^ This may be due to AKI has worsened the outcome of COVID-19 thus making survival very hard.

### Limitations of the study:

The limitations of current study were its relatively small sample size with retrospective design conducted at a single center. Still, this was the first study in Pakistan in which incidence of AKI in COVID-19 and its associated risk factors were determined. A prospective multi center-oriented study with larger sample size is needed for further evaluation.

## CONCLUSION

Half of the COVID-19 hospitalized patients developed AKI. Increasing age, prolonged hospital stays, hypoxemia, hypoalbuminemia, DM and raised inflammatory markers were the most significant risk factors for developing AKI.

### Authors’ Contributions:

**MA** gave main idea, developed conceptual frame work, did final write up and performed drafting of the work and revised it critically for important intellectual content. He is responsible and accountable for the accuracy and integrity of the work).

**OF** performed Literature review.

**MR** performed data Collection and analysis.

**AM** gave final approval of the version to be published..
